# Development and Validation of a Machine Learning Model to Explore Tyrosine Kinase Inhibitor Response in Patients With Stage IV *EGFR* Variant–Positive Non–Small Cell Lung Cancer

**DOI:** 10.1001/jamanetworkopen.2020.30442

**Published:** 2020-12-17

**Authors:** Jiangdian Song, Lu Wang, Nathan Norton Ng, Mingfang Zhao, Jingyun Shi, Ning Wu, Weimin Li, Zaiyi Liu, Kristen W. Yeom, Jie Tian

**Affiliations:** 1 Department of Biomedical Engineering, College of Medicine and Biological Information Engineering, Northeastern University. Shenyang, Liaoning, China; 2 Department of Radiology, School of Medicine Stanford University, Stanford, California; 3 Department of Medical Informatics, China Medical University, Shenyang, Liaoning, China; 4 Department of Medical Oncology, The First Hospital of China Medical University, Shenyang, Liaoning, China; 5 Department of Radiology, Shanghai Pulmonary Hospital, Shanghai, China; 6 National Cancer Center/Cancer Hospital, Chinese Academy of Medical Sciences and Peking Union Medical College, Beijing, China; 7 Department of Respiratory and Critical Care Medicine, West China Hospital, Chengdu, Sichuan, China; 8 Department of Radiology, Guangdong General Hospital, Guangdong Academy of Medical Sciences, Guangzhou, China; 9 Beijing Advanced Innovation Center for Big Data-Based Precision Medicine, School of Medicine and Engineering, Beihang University, Beijing, China

## Abstract

**Importance:**

An end-to-end efficacy evaluation approach for identifying progression risk after epidermal growth factor receptor (EGFR)–tyrosine kinase inhibitor (TKI) therapy in patients with stage IV *EGFR* variant–positive non–small cell lung cancer (NSCLC) is lacking.

**Objective:**

To propose a clinically applicable large-scale bidirectional generative adversarial network for predicting the efficacy of EGFR-TKI therapy in patients with NSCLC.

**Design, Setting, and Participants:**

This diagnostic/prognostic study enrolled 465 patients from January 1, 2010, to August 1, 2017, with follow-up from February 1, 2010, to June 1, 2020. A deep learning (DL) semantic signature to predict progression-free survival (PFS) was constructed in the training cohort, validated in 2 external validation and 2 control cohorts, and compared with the radiomics signature.

**Exposures:**

An end-to-end bidirectional generative adversarial network framework was designed to predict the progression risk in patients with NSCLC.

**Main Outcomes and Measures:**

The primary end point was PFS, considering the time from the initiation of therapy to the date of recurrence, confirmed disease progression, or death.

**Results:**

A total of 342 patients with stage IV *EGFR* variant–positive NSCLC receiving EGFR-TKI therapy met the inclusion criteria. Of these, 145 patients from 2 of the hospitals (n = 117 and 28) formed a training cohort (mean [SD] age, 61 [11] years; 87 [60.0%] female), and the patients from 2 other hospitals comprised 2 external validation cohorts (validation cohort 1: n = 101; mean [SD] age, 57 [12] years; 60 [59.4%] female; and validation cohort 2: n = 96, mean [SD] age, 58 [9] years; 55 [57.3%] female). Fifty-six patients with advanced-stage *EGFR* variant–positive NSCLC (mean [SD] age, 52 [11] years; 26 [46.4%] female) and 67 patients with advanced-stage *EGFR* wild-type NSCLC (mean [SD] age, 54 [10] years; 10 [15.0%] female) who received first-line chemotherapy were included. A total of 90 (26%) receiving EGFR-TKI therapy with a high risk of rapid disease progression were identified (median [range] PFS, 7.3 [1.4-32.0] months in the training cohort, 5.0 [0.6-34.6] months in validation cohort 1, and 6.4 [1.8-20.1] months, in validation cohort 2) using the DL semantic signature.The PFS decreased by 36% (hazard ratio, 2.13; 95% CI, 1.30-3.49; *P* < .001) compared with that in other patients (median [range] PFS, 11.5 [1.5-64.2] months in the training cohort, 10.9 [1.1-50.5] in validation cohort 1, and 8.9 [0.8-40.6] months in validation cohort 2. No significant differences were observed when comparing the PFS of high-risk patients receiving EGFR-TKI therapy with the chemotherapy cohorts (median PFS, 6.9 vs 4.4 months; *P* = .08). In terms of predicting the tumor progression risk after EGFR-TKI therapy, clinical decisions based on the DL semantic signature led to better survival outcomes than those based on radiomics signature across all risk probabilities by the decision curve analysis.

**Conclusions and Relevance:**

This diagnostic/prognostic study provides a clinically applicable approach for identifying patients with stage IV *EGFR* variant–positive NSCLC who are not likely to benefit from EGFR-TKI therapy. The end-to-end DL-derived semantic features eliminated all manual interventions required while using previous radiomics methods and have a better prognostic performance.

## Key Points

**Question:**

Can an end-to-end deep learning network model be used to identify patients with stage IV epidermal growth factor receptor (*EGFR*) variant–positive non–small cell lung cancer who will not benefit from EGFR–tyrosine kinase inhibitor (TKI) therapy?

**Findings:**

In this diagnostic/prognostic study of 342 patients receiving EGFR-TKI therapy, a bidirectional generative adversarial network model demonstrated a 36% reduction in the progression-free survival of patients at high risk for rapid progression but no significant difference in the progression-free survival between these patients and those receiving first-line chemotherapy. The proposed deep learning semantic signature eliminated all manual interventions required while using previous radiomics methods and had a better prognostic performance.

**Meaning:**

An end-to-end clinically applicable approach is promising for quantitatively identifying the benefit of EGFR-TKI therapy.

## Teaser

This diagnostic/prognostic study proposes a clinically applicable large-scale bidirectional generative adversarial network for predicting the efficacy of epidermal growth factor receptor–tyrosine kinase inhibitor therapy in patients with non–small cell lung cancer.

## Introduction

Epidermal growth factor receptor (EGFR)–tyrosine kinase inhibitor (TKI) therapy plays an important role in treating clinical stage IV *EGFR* (OMIM 131550) variant–positive non–small cell lung cancer (NSCLC).^1,2^ Previous studies^3,4^ have found that EGFR-TKIs, such as erlotinib, gefitinib, and icotinib, promote longer progression-free survival (PFS) in patients with *EGFR* variant–positive NSCLC than conventional chemotherapy. On the basis of these results, the current clinical guidelines recommend EGFR-TKI therapy in patients with stage IV *EGFR* variant–positive NSCLC.^5^

Despite potential benefits, studies^6,7^ have reported that 30% of patients with stage IV *EGFR* variant–positive NSCLC nevertheless developed rapid tumor progression after EGFR-TKI therapy, and TKIs were deemed ineffective or less effective in these patients.^4,8^ Currently, there are no known biomarkers predictive of which patients might not benefit from TKIs and are thus at risk for rapid disease progression. Next-generation TKIs (osimertinib) or intercalated therapy may be considered in patients with *EGFR* T790M variants or those more likely to develop tumor progression.^9,10,11^ Recent clinical studies^12,13,14^ have suggested that the efficacy of EGFR-TKIs should be evaluated before clinical decision-making and alternative treatments potentially prioritized in patients at high risk for rapid tumor progression.

However, in current clinical practice, no clear strategies exist for assessing future risk status after EGFR-TKI therapy in patients with stage IV *EGFR* variant–positive NSCLC. The primary method remains surveillance using chest computed tomography (CT) in search of macroscopic changes in disease. Recent studies^15,16^ have suggested potential utility of CT-based radiomics-derived predictors of prognosis in patients with stage IV *EGFR* variant–positive NSCLC. However, clinical translation of radiomics can be limiting because of multiple steps, such as manual segmentation of tumor boundaries, feature extraction, feature selection, and model development procedures.^17^ Bias might arise from interobserver variations in determining tumor boundaries and different methods used to decode radiomic features. Furthermore, layer-by-layer region of interest labeling is labor-intensive; thus, it is difficult to meet the clinical real-time requirements. A clinically applicable, end-to-end approach to more precisely stratify patients with stage IV *EGFR* variant–positive NSCLC who are likely responders and nonresponders to EGFR-TKI therapy could help to individualize care for this disease.

Recent developments in deep learning (DL) provide the potential to mitigate such clinical challenges.^18,19^ A generative adversarial network (GAN) has been reported to be useful in simulating real images and thereby bolstering learning image characteristics.^20^ Recent modifications to self-supervised representation learning with a GAN, the so-called large-scale bidirectional generative adversarial network (BigBiGAN), have resulted in state-of-the-art performances in generating high-quality, mimic images and automatically recognizing high-dimensional semantic features.^21^ Studies^22,23^ have found that semantic features extracted by the BigBiGAN framework can boost model performance in detecting coronavirus disease 2019 pneumonia and brain abnormalities.

We propose an end-to-end BigBiGAN-based DL approach to predicting the efficacy of EGFR-TKI therapy in patients presenting with stage IV *EGFR* variant–positive NSCLC without requiring manual tumor volume delineation or feature engineering procedures of traditional radiomics. We hypothesized that a DL-based representation framework could identify significant CT-based image features associated with disease progression in patients with stage IV *EGFR* variant–positive NSCLC and thereby contribute to identifying patient cohorts more likely to benefit from EGFR-TKI therapy.

## Methods

### Study Design

**Figure 1.**
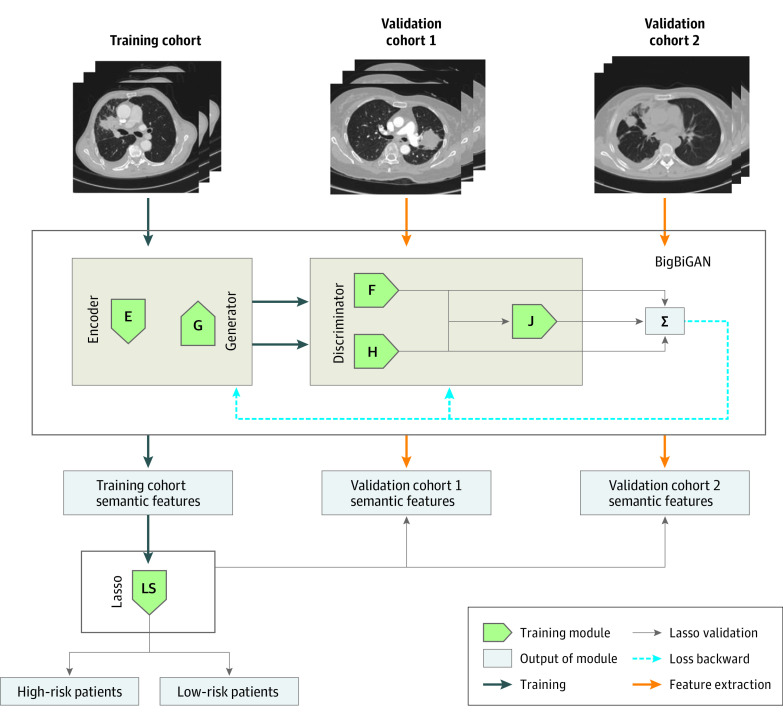
Flowchart of the Bidirectional Generative Adversarial Network (BigBiGAN) Training, Deep Learning Image Semantic Feature Extraction, and Construction and Validation of the Semantic Signature The training cohort was input into the encoder (E) module of the BigBiGAN for semantic feature extraction. The generator (G) module was used to mimic the original image from a random vector. Then the data pair of the original and mimic image was input into the F module of the discriminator, and the data pair of semantic features and random vector was input into the H module for loss calculation. When the loss of the model (J) was minimum, the semantic features of the training cohort were extracted for least absolute shrinkage and selection operator (LASSO) signature construction, and the signature was then tested on the 2 external validation cohorts. Σ indicates the sum of the loss.

The flowchart of this study is shown in Figure 1. First, 120-dimensional DL semantic features were extracted by the BigBiGAN framework to identify the CT features in individual patients with stage IV *EGFR* variant–positive NSCLC. Second, a semantic signature was proposed using the least absolute shrinkage and selection operator (LASSO) Cox proportional hazards regression method to distinguish the risk of progression after EGFR-TKI therapy in patients in the training cohort, which was subsequently validated in multiple external validation cohorts. Third, 2 control cohorts of patients with advanced-stage (stage III-IV) *EGFR* variant–positive and *EGFR* wild-type NSCLC who received first-line chemotherapy were respectively enrolled to compare survival benefits. This diagnostic/prognostic study was approved by the institutional review boards of the participating institutions, which waived the need for informed consent. All data were deidentified. This study followed the Transparent Reporting of a Multivariable Prediction Model for Individual Prognosis or Diagnosis (TRIPOD) reporting guidelines.^24^

### Patients

From January 1, 2010, to August 1, 2017, we retrospectively enrolled patients with clinical stage IV *EGFR* variant–positive NSCLC treated with EGFR-TKI therapy and patients with advanced-stage NSCLC treated with first-line chemotherapy (without EGFR-TKIs) from 6 hospitals. For the EGFR-TKI therapy cohorts, the inclusion criteria were as follows. Experienced practitioners (N.N.N., M.Z., J.S., N.W., W.L., and Z.L.) collected CT scans and clinical data from each participating hospital. Clinical data elements included sex, age, smoking status, performance status score, histopathologic subtype (adenocarcinoma, squamous cell carcinoma, or others), *EGFR* variant subtype, and the administered therapeutic regimen. Patients diagnosed with clinical stage IV NSCLC according to the American Joint Committee on Cancer guidelines were older than 20 years, had a confirmed *EGFR* variant (*EGFR19* exon deletion, exon 21 L858R substitution, or other *EGFR* variant subtype), and had received EGFR-TKI therapy according to current clinical guidelines. Patients with a history of systemic anticancer therapy for advanced disease or resection were excluded. Patients obtained contrast enhanced and/or noncontrast CT before EGFR-TKI administration. Detailed CT protocols from each included hospital are listed in eAppendix 1 in the Supplement. To reduce the impact of the variation in images from different sources of CT scanners, all input images were standardized (eAppendix 1 in the Supplement).

In addition, 2 control cohorts of patients treated with first-line chemotherapy (non–EGFR-TKI) were included according to the following criteria: patients had been diagnosed with clinical advanced-stage NSCLC, had the above-mentioned complete demographic characteristics and histopathologic subtype, and had an *EGFR* variant test result (*EGFR* variant–positive or *EGFR* wild-type NSCLC). All clinical treatments in this study were conducted on a voluntary basis.

The patients receiving EGFR-TKI therapy at 2 of the hospitals were used as the training cohort, and patients from 2 other hospitals served as the 2 external validation cohorts. The control cohorts of patients with advanced-stage *EGFR* variant–positive and *EGFR* wild-type NSCLC treated with first-line chemotherapy served to compare survival benefits.

The Response Evaluation Criteria in Solid Tumors 1.1 standard was used to determine tumor response on CT examinations. The primary end point in this study was PFS (time from the initiation of therapy to the date of recurrence); confirmed disease progression; or death. The occurrence of relapse or progression was defined as the development of a new lesion or progression of the primary lesion. Patients were censored at the date of death from other causes or at the last visit (for those who were living without documented disease progression).

### DL Semantic Features

The patient’s original whole CT images were used as the input for the BigBiGAN framework. As shown in Figure 1, the CT images were first input into the encoder module to learn the semantic characteristics of the images, and then, the generator module generated the mimic images. In this study, we used only CT slices that contained 1 or more lung tumors to reduce unnecessary learning. Second, the image data pair (real and generated images) and semantic feature data pair (learned from the real image and random) were input into the discriminator to calculate the loss of each module. By continuous self-supervised training of the BigBiGAN, 120-dimensional semantic features representative of the specific image characteristics were extracted from each CT image. The model was trained on the training cohort until the loss was minimum and kept stable. Then we used the model with the loss at the minimum state to test the 2 external validation cohorts. To ensure reproducibility, all the source codes are open access (eAppendix 1 in the Supplement) and implemented in the free Google Colab (Google Research).

### DL Semantic Signature

On the basis of the 120-dimensional semantic feature vector of each image obtained by the BigBiGAN model, for all CT slices from the same patient, we sequentially integrated the semantic feature vectors into a matrix. Finally, we calculated the mean value of each feature on all CT layers per patient. The averaged 120-dimensional semantic features of each patient in the training cohort were input into LASSO Cox proportional hazards regression to construct a semantic signature.

On the basis of the semantic signature score of each patient by LASSO, X-tile^25^ was used to obtain the optimal cutoff threshold to stratify the patients in the training cohort into the high-progression-risk group after EGFR-TKI therapy and the low-progression-risk group. Kaplan-Meier curves were plotted to present differences in PFS between different risk groups, and the log-rank test was used to evaluate differences between groups. Subsequently, the signature and the cutoff threshold were validated in the 2 external validation EGFR-TKI cohorts. Time-dependent receiver operating characteristic (ROC) curve analysis was used to evaluate the prognostic accuracy of the semantic signature in the 3 cohorts by the cutoffs of 10 and 12 months.

To further validate the semantic signature proposed in this study, we compared the radiomics prognostic signature for the efficacy evaluation of EGFR-TKIs. On the basis of radiomic features provided by the previous radiomics study,^15^ we compared the performance of the semantic signature proposed in this study with the radiomics signature in fitting the patients’ actual survival benefit. A more detailed introduction of the radiomics signature^15^ is presented in eAppendix 1 in the Supplement. The decision curve analysis and clinical impact curve analysis were used to evaluate the performances of the 2 signatures in actual clinical application.

### Statistical Analysis

Statistical analysis was performed using R software, version 3.2.3 (R Foundation for Statistical Computing). Independent, unpaired, 2-tailed *t* tests were used to compare the demographics of different groups for continuous variables. The Fisher exact test was used to identify associations between categorical variables. The PFS predicted using the semantic signature in the different progression risk groups was compared using hazard ratios (HRs). Time-dependent ROC curve analysis was performed using the package survivalROC. The decision curve analysis and clinical impact curve analysis results were plotted by dca.R and rmda, respectively. *P* < .05 (2-tailed) was considered statistically significant.

## Results

### Patients

A total of 342 patients with stage IV *EGFR* variant–positive NSCLC receiving EGFR-TKI therapy met the inclusion criteria. Of these, 145 patients from 2 of the hospitals (n = 117 and 28) formed a training cohort (mean [SD] age, 61 [11] years; 87 [60.0%] female), and the patients from 2 other hospitals comprised 2 external validation cohorts (validation cohort 1: n = 101; mean [SD] age, 57 [12] years; 60 [59.4%] female; and validation cohort 2: n = 96, mean [SD] age, 58 [9] years; 55 [57.3%] female). Fifty-six patients with advanced-stage *EGFR* variant–positive NSCLC (mean [SD] age, 52 [11] years; 26 [46.4%] female) and 67 patients with advanced-stage *EGFR* wild-type NSCLC (mean [SD] age, 54 [10] years; 10 [15.0%] female) who received first-line chemotherapy were included. The demographics and clinicopathologic data of the enrolled patients are presented in the Table. Detailed treatment scheme is presented in eAppendix 1 in the Supplement.

**Table.** Demographic and Histopathologic Characteristics of Study Patients^a^

Characteristic	EGFR-TKI therapy cohorts			Chemotherapy cohorts	
	Training cohort (n = 145)	Validation cohort 1 (n = 101)	Validation cohort 2 (n = 96)	*EGFR* variant positive (n = 56)	*EGFR* wild type (n = 67)
No. of CT sections	3481	1855	868	NA	NA
Sex					
Male	58 (40.0)	41 (40.6)	41 (42.7)	30 (53.6)	57 (85.1)
Female	87 (60.0)	60 (59.4)	55 (57.3)	26 (46.4)	10 (14.9)
Age, y					
≤65	84 (57.9)	66 (65.3)	66 (68.8)	43 (76.8)	60 (89.6)
>65	61 (42.1)	35 (34.7)	30 (31.2)	13 (23.2)	7 (10.4)
PS score					
≥2	100 (69.0)	68 (46.9)	62 (64.6)	43 (76.8)	39 (58.2)
<2	45 (31.0)	33 (53.1)	34 (35.4)	13 (23.2)	28 (41.8)
PFS, median (SD), mo	10.1 (15.0)	9.2 (9.0)	8.2 (7.9)	4.5 (4.7)	3.6 (3.6)
*EGFR* variant					
*EGFR* 19Del	72 (49.7)	60 (59.4)	41 (42.7)	21 (37.5)	NA
*EGFR* 21L858R	59 (40.7)	35 (34.6)	48 (50.0)	30 (53.6)	NA
Other	14 (9.6)	6 (6.0)	7 (7.3)	5 (8.9)	NA
Tobacco use					
Smoker	60 (41.3)	21 (20.8)	17 (17.7)	14 (25.0)	55 (82.1)
Nonsmoker	85 (58.7)	80 (79.2)	79 (82.3)	42 (75.0)	12 (17.9)
Histopathology					
Adenocarcinoma	135 (93.1)	99 (98.0)	92 (95.8)	31 (55.4)	12 (17.9)
SCC	9 (6.2)	1 (1.0)	3 (3.1)	25 (44.6)	55 (82.1)
Other	1 (0.7)	1 (1.0)	1 (1.1)	0	0

Abbreviations: EGFR, epidermal growth factor receptor; CT, computed tomography; NA, not applicable; PFS, progression-free survival; PS, performance status; SCC, squamous cell carcinoma; TKI, tyrosine kinase inhibitor.

^a^ Data are presented as number (percentage) of patients unless otherwise indicated.

The median (range) follow-up periods were 15.9 (1.7-68.0) months in the training cohort, 13.5 (1.5-57.0) months in external validation cohort 1, and 11.8 (2.0-55.0) months in external validation cohort 2, and 313 of 342 patients (91.5%) in the EGFR-TKI cohorts experienced tumor progression (29 patients, censored data). No significant difference in PFS among the 3 cohorts (median [range] PFS, 9.9 [1.4-64.2] months in the training cohort, 9.2 [0.6-50.5] months in validation cohort 1, and 8.2 [0.8-40.6] months in validation cohort 2; Kruskal-Wallis H test, *P* = .20) was found. Furthermore, no significant differences in patient demographics among all 3 cohorts presented in the Table were found. Two patients receiving chemotherapy were censored during the follow-up; in 1 case, treatment was discontinued because of serious adverse reactions. The median (range) PFS were 3.6 (1.0-16.3) in months for *EGFR* wild-type NSCLC and 4.5 (0.9-25.9) months in *EGFR* variant-positive NSCLC.

### DL Semantic Features and Signature

A total of 6204 CT sections that contained lung tumor(s) from the 342 patients in the EGFR-TKI therapy cohorts were included. In the last epoch of the training of the BigBiGAN framework, we extracted the semantic features when the network loss was minimum.

**Figure 2.**
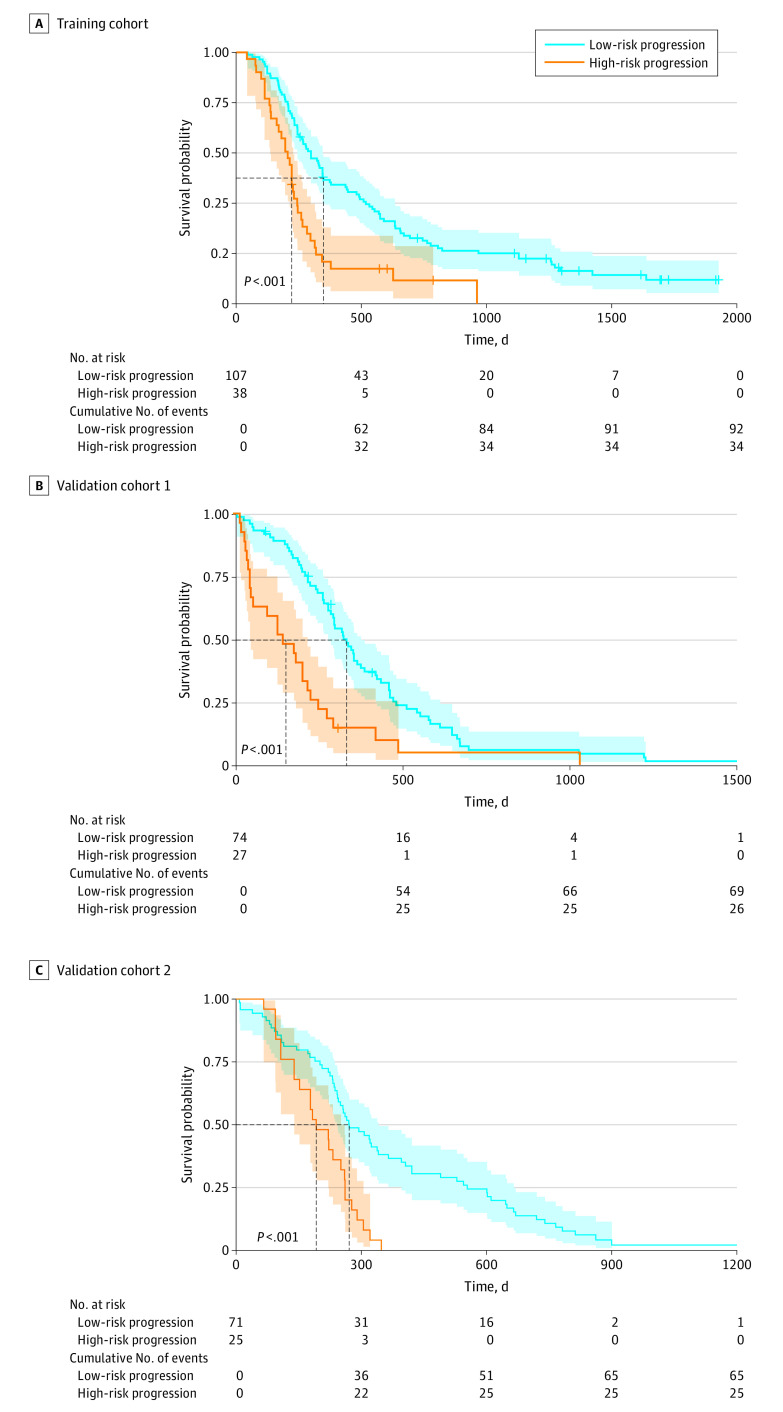
Kaplan-Meier Curves of Patients in the 3 Epidermal Growth Factor Receptor–Tyrosine Kinase Inhibitor Therapy Cohorts Stratified as High Risk and Low Risk of Rapid Progression Using the Deep Learning Semantic Signature Proposed in this Study Shaded areas indicate 95% CIs.

The 145 × 120–dimensional feature matrix obtained from the training cohort was input into the LASSO Cox proportional hazards regression model to construct the semantic prognostic signature. The coefficients of the 18 significant semantic features calculated by LASSO Cox proportional hazards regression are listed in eAppendix 2 in the Supplement. On the basis of the semantic signature score calculated for the patients in the training cohort, a cutoff threshold of −0.67 was obtained by X-tile to stratify the patients into low-progression-risk (107 patients; median [range] PFS, 11.5 [1.5-64.2] months) and high-progression-risk (38 patients; median [range] PFS, 7.3 [1.4-32.0] months) groups. A significant difference in PFS was found between the 2 groups (HR, 2.13; 95% CI, 1.30-3.49; *P* < .001). The Kaplan-Meier curves are presented in Figure 2A.

When applying the signature to the external validation cohorts, the median (range) PFS was 10.9 (1.1-50.5) months for the low-risk group (74 patients) and 5.0 (0.6-34.6) months for the high-risk group (27 patients) in validation cohort 1. Significant differences in PFS were found between the 2 risk groups (HR, 2.35; 95% 15 CI, 1.31–4.21; *P* < .001). Regarding validation cohort 2, a significant PFS difference was found between the low-risk (71 patients; median [range], 8.9 [0.8-40.6] months) and high-risk (25 patients; median [range], 6.4 [1.8-20.1] months) groups (HR, 2.32; 95% CI, 1.28–4.20; *P* < .001). The Kaplan-Meier curves are presented in Figure 2B and C.

Time-dependent ROC analysis indicated that the area under the curves of the survival benefits predicted by the semantic signature in the training cohort were 0.78 (95% CI, 0.75-0.80) under the 12-month cutoff and 0.75 (95% CI, 0.71-0.79) under the 10-month cutoff. The prediction area under the curves in external validation cohort 1 was 0.73 (95% CI, 0.70-0.77) under the 12-month cutoff and 0.72 (95% CI, 0.68-0.76) under the 10-month cutoff. In external validation cohort 2, the prediction area under the curves was 0.75 (95% CI, 0.72-0.80) under the 12-month cutoff and 0.70 (95% CI, 0.66-0.74) under the 10-month cutoff (eFigure 1 in the Supplement).

**Figure 3.**
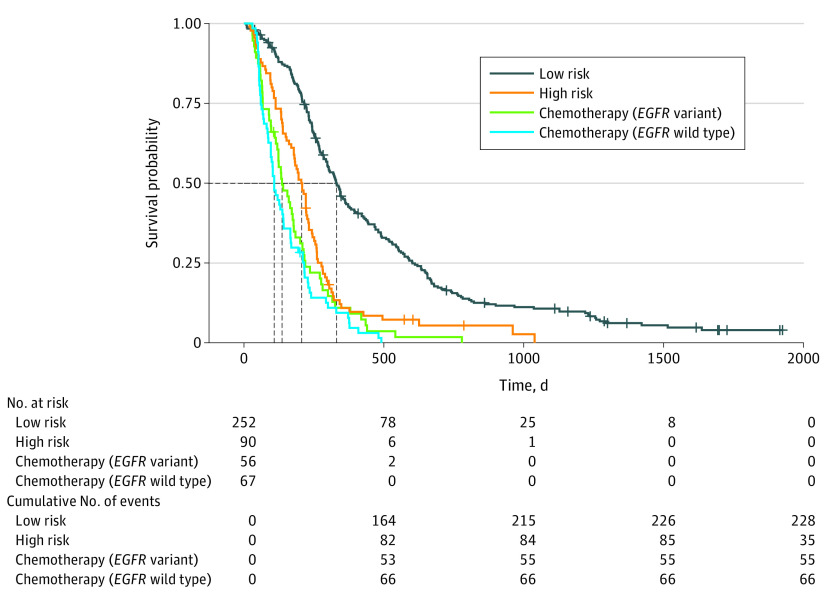
Kaplan-Meier Curves of Patients With Low Progression Risk and High Progression Risk Who Received Epidermal Growth Factor Receptor–Tyrosine Kinase Inhibitor and Patients With *EGFR* Variant–Positive and *EGFR* Wild-Type Non–Small Cell Lung Cancer Who Received First-Line Chemotherapy The dotted lines represent the median time of progression-free survival of patients in each cohort.

The results indicated that the median (range) PFS was 10.8 (0.8-64.2) months for the low-progression-risk group, 6.9 (0.6-34.6) months for the high-progression-risk group, 4.5 (0.9-25.9) months for the *EGFR* variant–positive chemotherapy group, and 3.6 (1.0-16.3) months for the *EGFR* wild-type chemotherapy group. No significant differences in PFS were found between the high-progression-risk patients in the EGFR-TKI cohort and the patients with *EGFR* variant–positive or *EGFR* wild-type NSCLC receiving chemotherapy (median PFS, 6.9 vs 4.4 months; *P* = .08). However, the survival benefit was significantly better in the low-progression-risk patients in the EGFR-TKI cohort than in the other 3 groups (median PFS, 10.8 vs 4.4 months; *P* < .001) (Figure 3). A more detailed comparison of these 4 groups is presented in eAppendix 3 in the Supplement.

### Comparison With Radiomics Signatures

**Figure 4.**
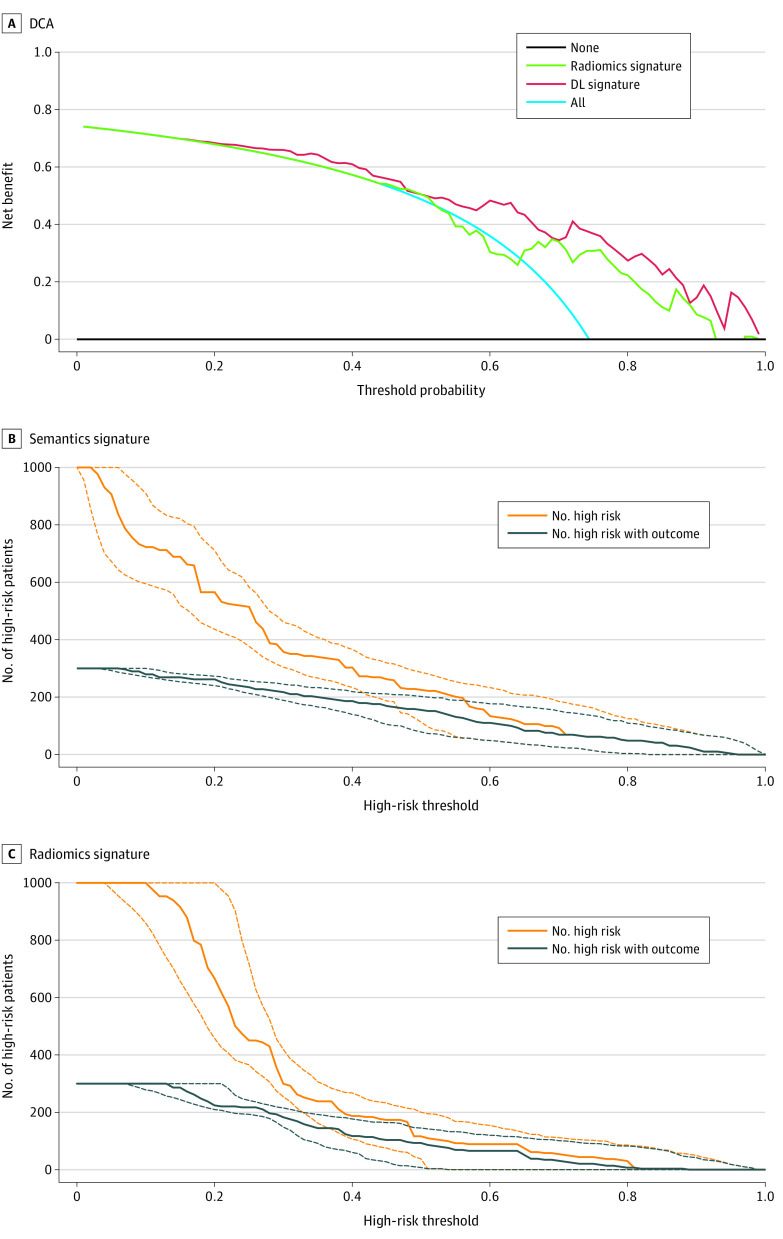
Decision Curve Analysis (DCA) and Clinical Impact Curve Analysis (CIA) The orange line in the CIA plots indicates the number of patients predicted by deep learning (DL) to be at high risk for disease progression, and the blue line indicates the actual number at high risk for progression. The prediction result of the DL signature is consistent with that of patients who actually progressed when the high-risk probability was higher than 0.7.

The decision curve analysis indicated that when the semantic signature is used to predict the risk of progression after EGFR-TKI therapy, patients could obtain better clinical benefits across all risk probabilities (Figure 4A). Furthermore, the comparison of clinical impact curves between the 2 signatures indicated that the predictions by the semantic signature of which patients were at high risk for progression were consistent with who actually experienced disease progression when the risk threshold was 0.7; this value was 0.8 for the radiomics signature (Figure 4B and C).

## Discussion

This diagnostic/prognostic study examined an end-to-end, CT-based DL approach to predict efficacy of EGFR-TKI therapy in patients with stage IV *EGFR* variant–positive NSCLC based on disease course. The study used a state-of-the-art representation learning framework to build a DL semantic prognosis signature from pretherapy CT images of patients with NSCLC. The proposed semantic signature suggested that 30% of patients were less likely to benefit from EGFR-TKI therapy. Patients with CT-based semantic features that were considered low risk for progression (74% of all patients) had a median (range) PFS of 327 (0.8-64.2) days compared with those with high-risk sematic features, who demonstrated a median (range) PFS of 208 (0.6-34.6) days. In addition, no significant difference in survival was found between patients with high-risk semantic features and who received first-line chemotherapy (without EGFR-TKI therapy: median PFS, 6.9 vs 4.4 months; *P* = .08).

Compared with radiomics, the approach used in this study not only avoids human intervention procedures requiring manual region of interest segmentation and predesigning heterogeneous features, but it also appears to be associated with better survival prognostic performance. Finally, the open-access source code provided in this study enables interested readers to reproduce the results of this study.

In a recent study^8^ on EGFR-TKI therapy, approximately 30% of patients failed to benefit from TKI drugs, with earlier disease progression in these patients. On the basis of DL semantic signature, patients predicted to have high risk of rapid progression accounted for 26% of all patients and a PFS that was similar to patients with advanced-stage NSCLC who received conventional chemotherapy. Furthermore, the clinical impact curve analysis curves indicated that when using the DL semantic signature to identify patients at high risk of progression, the predicted result was consistent with the patient’s actual tumor progression when the probability was higher than 0.7. This result had good agreement with the report of previous clinical studies,^8,15^ in which 30% of patients had rapid progression after EGFR-TKI therapy. These findings suggest potential use of a DL-based framework for more precisely stratifying patients with NSCLC who are more likely to benefit from EGFR-TKI therapy.

There have been many challenges to developing clinically applicable tools to evaluate the efficacy of EGFR-TKI therapy.^26^ Although potential image-based methods have been reported,^15,16,26,27^ important hurdles that relate to clinical operability and real-time requirements in clinics exist. Manual delineation of tumor boundary can create bias and can be labor-intensive, especially with multiple lung lesions.^28^ In this study, a DL-based approach was used to identify significant semantic features without requiring tumor margin delineation. Thus, unlike the more labor-intensive radiomics-based image predictors, an end-to-end DL approach to generate CT-based biomarkers to stratify patients who are more likely to benefit from EGFR-TKI therapy can facilitate more rapid clinical translation. Direct comparisons between the image features extracted by DL networks and those extracted by radiomics are currently lacking. These results also suggest that DL-based semantic features achieve better performance than radiomics in predicting the efficacy of EGFR-TKI therapy.

Chemotherapy remains the mainstay therapy for advanced-stage *EGFR* wild-type NSCLC. In this study, one potential reason for worse PFS in the *EGFR* wild-type group may be the rate of squamous cell carcinoma pathology, which was 82% in this group. Prior studies^29,30^ have suggested that the efficacy of chemotherapy in patients with lung adenocarcinoma is superior to that in patients with other pathologic subtypes. However, studies^31,32^ have not found this to be the case for EGFR-TKI therapy, prompting a clinical need to better stratify patients with *EGFR* variant–positive NSCLC for EGFR-TKI therapy efficacy.

### Limitations

This study has limitations. The efficacy of osimertinib was much better than that of previous drugs, such as erlotinib, gefitinib, and icotinib, in this study. More patients receiving osimertinib should be included in future studies to clarify the risk of progression according to different TKI drugs. In addition, this study used CT images that only included lung tumors to train the model and thus reduce unnecessary calculations; therefore, screening of CT sections is needed. Also, there is currently no specific definition of the DL semantic features, and we will further explore the image encoding process corresponding to the DL semantic features to explain the specific image characteristics reflected by these features.

## Conclusions

We describe an end-to-end CT-based DL approach to stratify patients who are more likely to benefit from EGFR-TKI therapy and contribute to precision in therapeutics in NSCLC. Unlike the more labor-intensive radiomics-based image predictors, an end-to-end DL approach to generate CT-based biomarkers can help toward more rapid clinical translation.

## Notes

Supplement. **eAppendix 1.** Open Access Source Code, CT Protocols of the Included Hospitals, Treatment Details and Follow-up, and the Radiomics Signature We Used for Comparison **eAppendix 2.** Patient Enrollment and Construction and Performance of the Signature **eAppendix 3.** Comparison of EGFR-TKI and Chemotherapy
