# Error in Author Affiliation

**DOI:** 10.1001/jamanetworkopen.2021.1634

**Published:** 2021-02-16

In the Original Investigation published December 17, 2020, “Development and Validation of a Machine Learning Model to Explore Tyrosine Kinase Inhibitor Response in Patients With Stage IV *EGFR* Variant–Positive Non–Small Cell Lung Cancer,”^1^ Dr Song’s affiliation was incorrect. He is affiliated with the School of Medical Informatics, China Medical University, Shenyang, Liaoning, China. This article has been corrected.^1^
