# Supplementary material for: Development and Validation of a Machine Learning Model to Explore Tyrosine Kinase Inhibitor Response in Patients With Stage IV EGFR Variant–Positive Non–Small Cell Lung Cancer
Source: JAMA Netw Open. 2020 Dec 17;3(12):e2030442. doi: 10.1001/jamanetworkopen.2020.30442 (PMC7747022; doi:10.1001/jamanetworkopen.2020.30442)
Supplement: Supplement. — eAppendix 1. Open Access Source Code, CT Protocols of the Included Hospitals, Treatment Details and Follow-up, and the Radiomics Signature We Used for Comparison eAppendix 2. Patient Enrollment and Construction and Performance of the Signature eAppendix 3. Comparison of EGFR-TKI and Chemotherapy [file jamanetwopen-e2030442-s001.pdf]

Supplementary Online Content

Song J, Wang L, Ng NN, et al. Development and validation of a machine learning model to explore tyrosine kinase inhibitor response in patients with stage IV *EGFR* variant–positive non–small cell lung cancer. *JAMA Netw Open*. 2020;3(12):e2030442. doi:10.1001/jamanetworkopen.2020.30442

eAppendix 1. Open Access Source Code**,** CT Protocols of the Included Hospitals, Treatment Details and Follow-up, and the Radiomics Signature We Used for Comparison

eAppendix 2. Patient Enrollment and Construction and Performance of the Signature

eAppendix 3. Comparison of EGFR-TKI and Chemotherapy

This supplementary material has been provided by the authors to give readers additional information about their work.

© 2020 Song J et al. *JAMA Network Open.*

eAppendix 1. Open Access Source Code, CT Protocols of the Included Hospitals, Treatment Details and Follow-up, and the Radiomics Signature We Used for Comparison

Open access source code

We used the publicly accessible BigBiGAN framework provided by TensorFlow Hub available at: https://tfhub.dev/deepmind/bigbigan-resnet50/1. Additionally, the publicly available Google Colab computing platform was used for program execution. The source code of this study is available at: https://github.com/JD910/EGFR-TKI-BBG-Training. The batch size and epoch of the BigBiGAN were set at 20 and 200 in this study. After training the framework, CT images in the two external validation cohorts were input into the model to extract the DL semantic features.

CT protocols of the included hospitals

Training cohort

Hospital 1: 1-3mm slice thickness with or without contrast CT scans were acquired on Philips Brilliance 40 and Siemens Defintion AS. Filter sharp (C) for CT reconstruction, while the Siemens Defination AS is with the following acquisition parameters: tube voltage = 120 kV, tube current = 130 mA, rotation time = 0.5s, detector collimation = 64 × 0.625 mm, FOV = 300 × 300 mm, image matrix = 512 × 512, kernel B31f medium sharp + for CT reconstruction.

Hospital 2: 1-3mm slice thickness with or without contrast (Lightspeed VCT and Revolution, GE Healthcare, Milwaukee, WI; Aquilion, Toshiba Medical Systems, Otawara, Japan; SOMATOM, Siemens, Erlangen, Germany).

© 2020 Song J et al. *JAMA Network Open.*

External validation cohort 1: Both chest non-enhanced and contrast-enhanced CT were performed on every patient using one of the two multi-detector row CT (MDCT) systems (GE Lightspeed Ultra 8, GE Healthcare, Hino, Japan or 64-slice LightSpeed VCT, GE Medical systems, Milwaukee, Wisconsin). All CT images were reconstructed with the standard kernel.

External validation cohort 2: 1-3mm slice thickness with or without contrast CT scans were obtained with SIEMENS SOMATOM Definition Flash scanners (Munich, Germany). The following parameters were used to obtain HRCT images: collimator with 64 × 0.6 mm, section thickness of 1 mm, reorganization interval of 0.66 mm, and tube voltage of 120 kV.

In order to reduce the impact of the variation in images from different sources on the learning efficiency of BigBiGAN, the following image standardization was performed to all the input images.

StandardScaler: *data_stand = (data - np.mean(data)) / np.std(data)*

Treatment details and follow-up

Erlotinib (n=67), gefitinib (n=194), icotinib (n=51), afatinib (n=12), and osimertinib (n=18) were administered to patients in the three EGFR-TKI cohorts. Three patients in the training cohort were diagnosed with advanced NSCLC when they were first admitted to our hospital and then treated with EGFR-TKI therapy. The clinical treatments provided in the chemotherapy cohorts were as follows: of 72 patients with pathologically confirmed SCC, 39 received gemcitabine plus cisplatin, 10 received docetaxel plus carboplatin, 11 received paclitaxel plus cisplatin, and 12 received docetaxel plus cisplatin. Fifty-one patients with pathologically confirmed adenocarcinoma received

© 2020 Song J et al. *JAMA Network Open.*

bevacizumab/pemetrexed plus carboplatin. All drug doses were administered in accordance with the current clinical guidelines and the patient's condition.

The averaged follow-up interval was 4–6 weeks in patients treated with EGFR-TKI therapy. Asymptomatic patients were followed up every 6 weeks, imaging was performed every 8-12 weeks, and for symptomatic patients more flexible and frequent follow up plan were developed. Patients treated with chemotherapy were reviewed every 3 weeks on average.

The radiomics signature we used for comparison

A prognostic radiomic signature which previously reported for EGFR-TKI efficacy prediction was used for comparison (please see the reference [16] in the manuscript). In order to construct this signature, 1032 phenotypic features were designed to be automatically extracted from the manually segmented tumor region for each patient. All the features were grouped by: 3D, texture, Gabor, and wavelet features that covered one-, two- and three-dimensional features. Then, 12 differently expressed radiomic phenotypic descriptors and their corresponding weights were obtained from the feature set in the training cohort for prognostic prediction by using the LASSO Cox proportional hazards regression. The established signature was applied to stratify the training cohort into slow- and rapid-progression subgroups of *EGFR* inhibitor, which was achieved by using the X-tile. The signature is presented as following.

Signature = (2.231 × 10^(-8) × value of "Contrast of Co-occurrence on LL in the 0° direction")

+ (7.590 × 10^(-4) × value of "Maximum-Probability of Co-occurrence on LL in the 0° direction")

+ (3.034 × 10^(-5) × value of "Maximum-Probability of Co-occurrence on LL in the 45° direction")

+ (5.353 × 10^(-5) × value of "Maximum-Probability of Co-occurrence on HL in the 0° direction")

© 2020 Song J et al. *JAMA Network Open.*

+ (1.010 × 10^(-4) × value of “Maximum-Probability of Co-occurrence on HL in the 45° direction”)

+ (4.482 × 10^(-6) × value of “Long-Run-High-Gray-Level Emphasis of Run Length on HL”)

+ (0.023 × value of “Entropy of GPTR in the 225° direction by two pixel steps”)

+ (0.116 × value of “Entropy of GPTR in the 45° direction by four pixel steps”)

– (1.324 × 10^(-7) × value of “Variance of GMTR in the 90° direction by four pixel steps”)

+ (0.115 × value of “Entropy of GPTR in the 135° direction by four pixel steps”)

– (9.290 × 10^(-9) × value of “Variance of GMTR in the 225° direction by five pixel steps”)

+ (2.300 × 10^(-6) × value of “Maximum diameter of tumor”).

By using the radiomics signature, the difference in PFS between the high-risk patients and low-risk patients was significant ($P < 0.0001$). The area under curve of the time-dependent ROC curves for 10-month PFS prediction was 0.711 to 0.738, and that of for one-year PFS prediction was 0.701 to 0.822.

© 2020 Song J et al. *JAMA Network Open.*

eAppendix 2. Patient Enrollment and Construction and Performance of the Signature

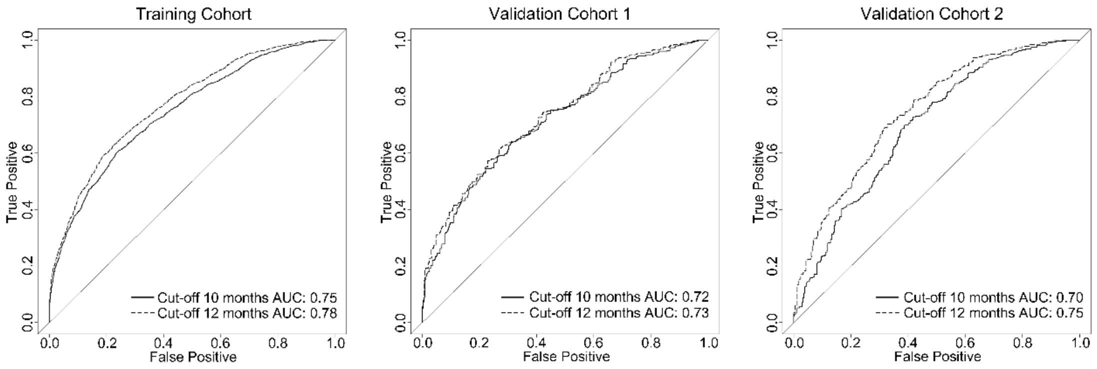

eFigure 1. Time-dependent receiver operating characteristic curves assessing the semantic signature for the training and two external validation EGFR-TKI cohorts. AUC: area under curve

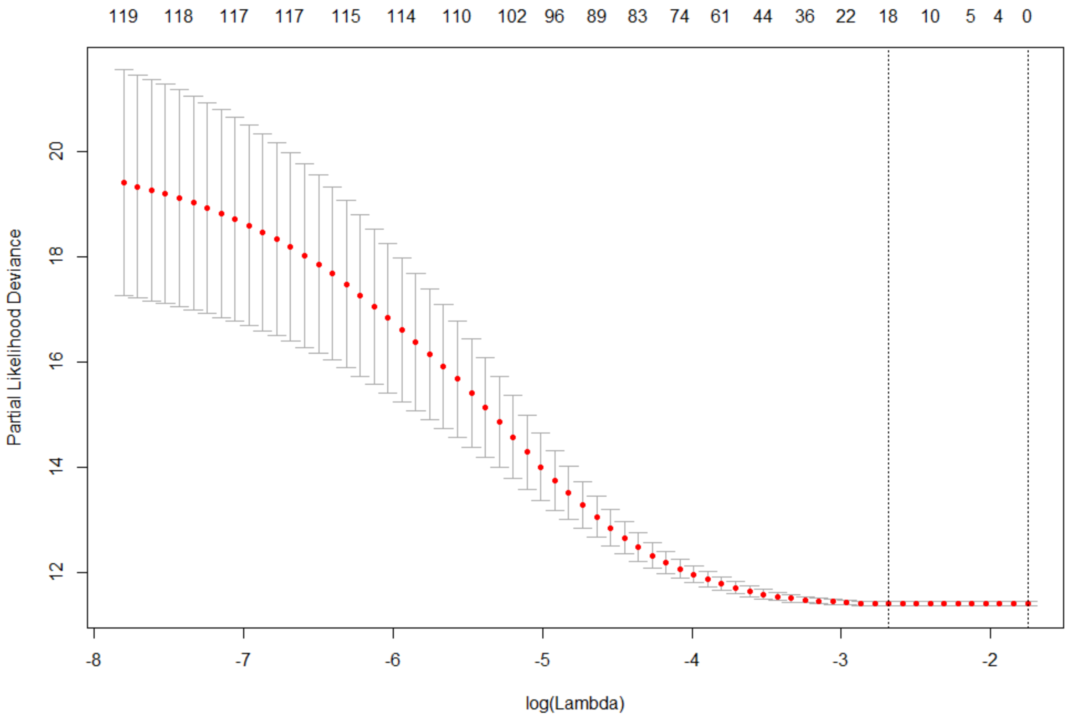

eFigure 2. Lasso Cox regression for deep learning semantic feature selection. The cv.glmnet() function was used for cox regression analysis, and the model at lambda.min was used for deep learning semantic signature construction.

© 2020 Song J et al. *JAMA Network Open.*

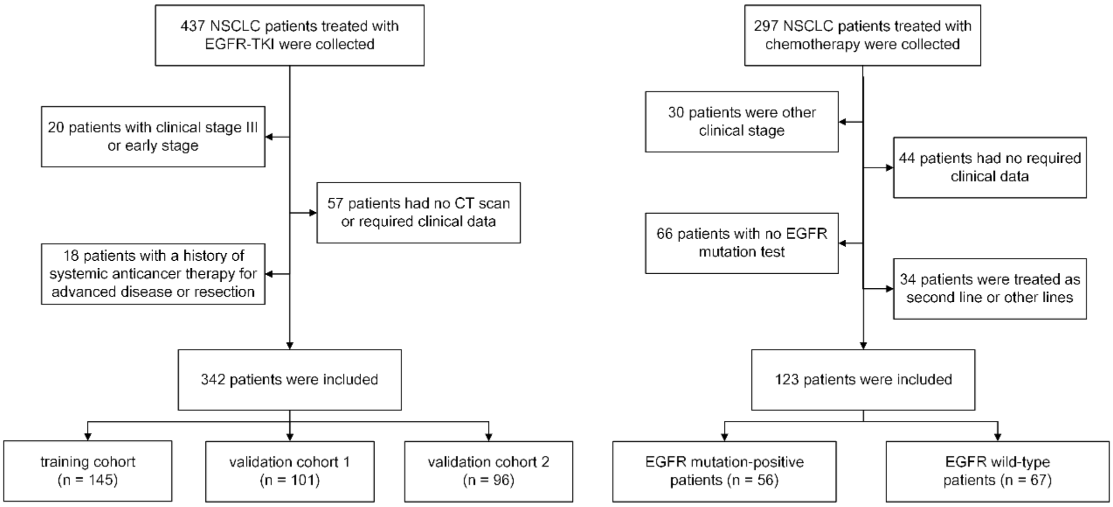

eFigure 3. Flowchart of patient enrollment in this study. NSCLC: non-small cell lung cancer, EGFR: epidermal growth factor receptor, TKIs: tyrosine kinase inhibitor

eTable 1. The 18 features and the corresponding coefficients by Lasso Cox. "." represents zero.

Feature	Coefficient
Feature1	-0.10814
Feature2	.
Feature3	.
Feature4	.
Feature5	.
Feature6	.
Feature7	.
Feature8	0.028038
Feature9	.
Feature10	.
Feature11	.
Feature12	.
Feature13	.
Feature14	.
Feature15	.
Feature16	.
Feature17	.
Feature18	.
Feature19	.
Feature20	.
Feature21	.
Feature22	.
Feature23	.
Feature24	.
Feature25	.
Feature26	-0.01804
Feature27	.
Feature28	.
Feature29	.
Feature30	-0.10374
Feature31	0.077858
Feature32	.
Feature33	.
Feature34	-0.00583
Feature35	.
Feature36	.
Feature37	.
Feature38	.
Feature39	.
Feature40	.

© 2020 Song J et al. *JAMA Network Open.*

Feature41	.
Feature42	.
Feature43	.
Feature44	.
Feature45	-0.03115
Feature46	.
Feature47	-0.13618
Feature48	.
Feature49	.
Feature50	.
Feature51	.
Feature52	.
Feature53	.
Feature54	.
Feature55	0.086864
Feature56	.
Feature57	.
Feature58	.
Feature59	.
Feature60	.
Feature61	.
Feature62	-0.01236
Feature63	.
Feature64	.
Feature65	.
Feature66	0.10337
Feature67	.
Feature68	.
Feature69	.
Feature70	.
Feature71	.
Feature72	.
Feature73	.
Feature74	.
Feature75	.
Feature76	.
Feature77	.
Feature78	.
Feature79	.
Feature80	.
Feature81	.
Feature82	.
Feature83	.

© 2020 Song J et al. *JAMA Network Open.*

Feature84	0.082194
Feature85	.
Feature86	.
Feature87	.
Feature88	.
Feature89	-0.21359
Feature90	.
Feature91	.
Feature92	.
Feature93	.
Feature94	.
Feature95	.
Feature96	.
Feature97	.
Feature98	-0.02298
Feature99	-0.02462
Feature100	.
Feature101	0.090777
Feature102	.
Feature103	.
Feature104	.
Feature105	.
Feature106	.
Feature107	.
Feature108	.
Feature109	.
Feature110	.
Feature111	-0.13377
Feature112	.
Feature113	.
Feature114	.
Feature115	-0.01575
Feature116	.
Feature117	.
Feature118	.
Feature119	.
Feature120	.

Appendix 3. Comparison of EGFR-TKI and Chemotherapy

Comparison of EGFR-TKI and chemotherapy

To further clarify the difference in PFS in patients at high-risk of rapid progression, and patients at low-risk of rapid progression, and patients in the two chemotherapy cohorts, we conducted two comparative experiments in this study.

Comparison 1: We first integrated the patients in the three EGFR-TKI cohorts, and divided them into low-progression-risk and high-progression-risk groups in accordance with the Lasso signature. Then, the PFS of patients in the four groups were compared, as described in the manuscript.

Comparison 2: We removed the training cohort, and only the patients in the two external validation cohorts were integrated and divided into low-progression-risk and high-progression-risk groups in accordance with the proposed Lasso signature. Then, the PFS of patients in the four groups were compared.

The results of the above comparisons are presented as following.

eTable 2. Statistical data of the Comparison 1.

	Number	Median PFS	HRs (95% CI), P value	HRs (95% CI), P value
EGFR TKI				
Low-progression-risk	252	10.8	Ref	-
High-progression-risk	90	6.9	0.48 (0.36–0.64), P<0.0001	Ref
Chemotherapy				
EGFR mutation-positive	56	4.7	0.35 (0.24–0.52), P<0.0001	0.72 (0.45–1.13), P>0.05
EGFR wild-type	67	3.6	0.28 (0.19–0.42), P<0.0001	0.68 (0.47–1.03), P>0.05

eTable 3. Statistical data of the Comparison 2.

	Number	Median PFS	HRs (95% CI), P value	HRs (95% CI), P value
EGFR TKI				

Low-progression-risk	145	10.0	Ref	-
High-progression-risk	52	6.1	0.46 (0.33–0.66), *P*<0.0001	Ref
Chemotherapy				
EGFR mutation-positive	56	4.7	0.41 (0.28–0.59), *P*<0.0001	0.88 (0.55–1.41), *P*>0.05
EGFR wild-type	67	3.6	0.33 (0.23–0.48), *P*<0.0001	0.71 (0.44–1.15), *P*>0.05

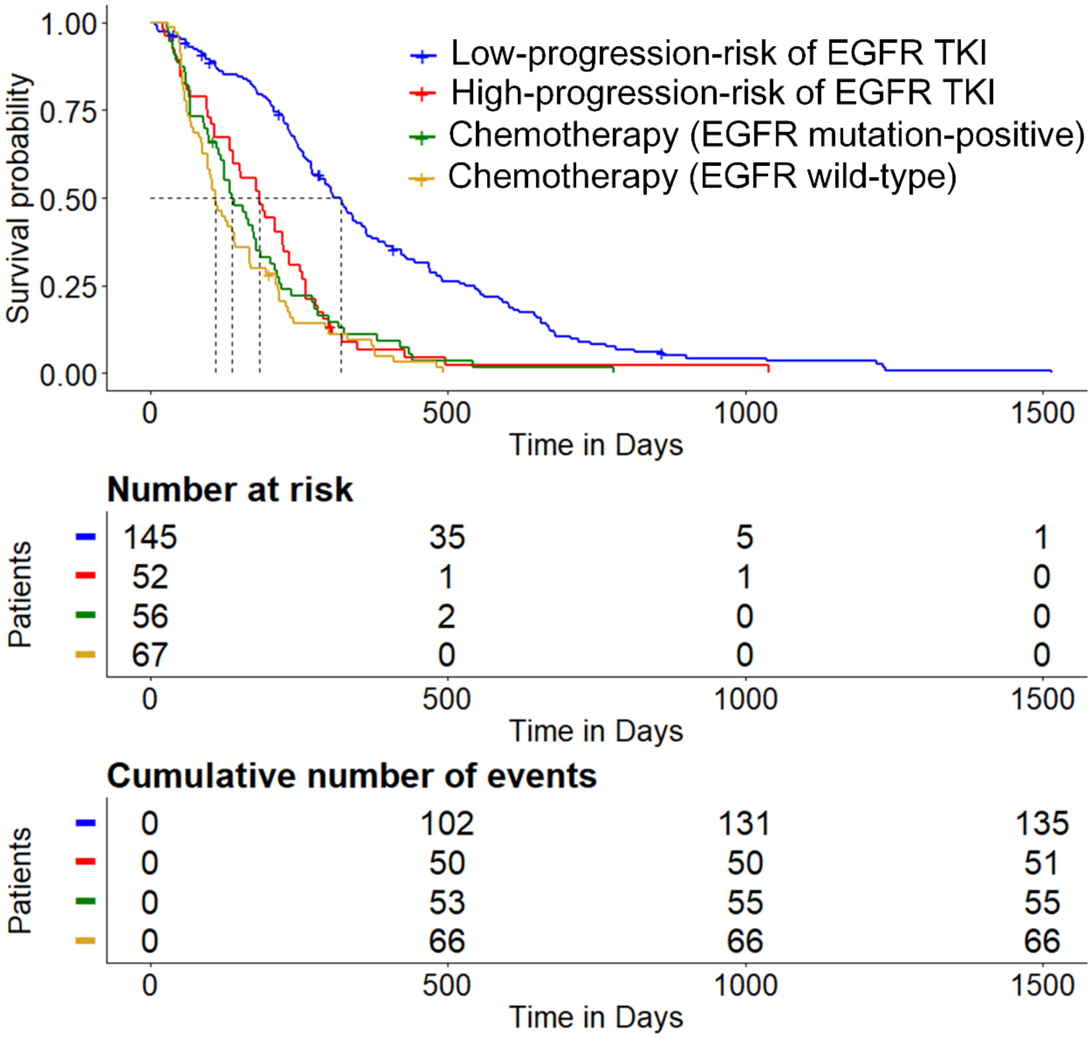

eFigure 4. Results of the comparison without the training cohort. Kaplan-Meier curves of the low-progression-risk (blue) and high-progression-risk (red) patients who received EGFR-TKI therapy, and patients with *EGFR* mutation-positive (green) and *EGFR* wild-type (yellow) who received first-line chemotherapy. The dotted lines represent the median PFS of patients in each cohort. EGFR: epidermal growth factor receptor; TKIs: tyrosine kinase inhibitor; PFS: progression-free survival

© 2020 Song J et al. *JAMA Network Open.*
